# Drought Stress Induces Morpho-Physiological and Proteome Changes of *Pandanus amaryllifolius*

**DOI:** 10.3390/plants11020221

**Published:** 2022-01-15

**Authors:** Muhammad Asyraf Mohd Amnan, Wan Mohd Aizat, Fiqri Dizar Khaidizar, Boon Chin Tan

**Affiliations:** 1 Centre for Research in Biotechnology for Agriculture (CEBAR), Universiti Malaya, Kuala Lumpur 50603, Malaysia; m.asyraf.amnan@um.edu.my (M.A.M.A.); fiqri@um.edu.my (F.D.K.); 2 Institute of Systems Biology (INBIOSIS), Universiti Kebangsaan Malaysia, Bangi 43600, Malaysia; wma@ukm.edu.my

**Keywords:** antioxidant enzymes, drought stress, *Pandanus amaryllifolius*, proteomics, stress-responsive proteins, TMT-labelled LCMS/MS

## Abstract

Drought is one of the significant threats to the agricultural sector. However, there is limited knowledge on plant response to drought stress and post-drought recovery. *Pandanus amaryllifolius*, a moderate drought-tolerant plant, is well-known for its ability to survive in low-level soil moisture conditions. Understanding the molecular regulation of drought stress signaling in this plant could help guide the rational design of crop plants to counter this environmental challenge. This study aimed to determine the morpho-physiological, biochemical, and protein changes of *P. amaryllifolius* in response to drought stress and during recovery. Drought significantly reduced the leaf relative water content and chlorophyll content of *P. amaryllifolius*. In contrast, relative electrolyte leakage, proline and malondialdehyde contents, and the activities of antioxidant enzymes in the drought-treated and recovered samples were relatively higher than the well-watered sample. The protein changes between drought-stressed, well-watered, and recovered plants were evaluated using tandem mass tags (TMT)-based quantitative proteomics. Of the 1415 differentially abundant proteins, 74 were significantly altered. The majority of proteins differing between them were related to carbon metabolism, photosynthesis, stress response, and antioxidant activity. This is the first study that reports the protein changes in response to drought stress in *Pandanus*. The data generated provide an insight into the drought-responsive mechanisms in *P. amaryllifolius*.

## 1. Introduction

Drought stress is a significant threat to agricultural productivity worldwide, causing 83% of agricultural economic losses. About US$29 billion was lost from all combined agriculture damages due to natural disasters [1]. Drought has affected the rice grain yield and caused about US$840 million losses in several rice-producing regions in Thailand [2]. In Malaysia, a 12–51% reduction in rice yield due to drought stress was reported from 2007 to 2011 [3]. Moreover, an expanding world population increases pressure on agriculture to use water more efficiently. Hence, it is indispensable to understand the drought response and adaptive mechanisms of plants as it could help to improve crop performance under drought stress conditions.

Drought stress disturbs physiological and biochemical processes in plants, including cell membrane, disrupting transportation of solutes, photosynthesis rate, nutrient uptake, translocation, and causes electron leakage and excessive accumulation of reactive oxygen species (ROS) [4,5]. The impacts of drought stress on plants rely on the severity and the growth period of plants [6]. To cope with these adverse effects, plants have developed intricate responses and adaptive strategies. These include the overproduction of compatible osmolytes, alteration of endogenous hormonal levels, and regulation of physiological and molecular changes [7]. Under moderate drought conditions, plants respond and adapt by altering root architecture [8] and stomatal closure [9], hoping to maintain a balance between stress tolerance and growth. However, if drought conditions become severe, plants tend to activate protection mechanisms against cellular damage, adjust in vivo antioxidant enzyme systems to eliminate excessive ROS, and accumulate proteins to maintain cell turgor, aiming to survive under such conditions [9].

Extensive efforts have been made to understand the plant responsive and adaptive mechanisms to drought stress using different “omics” techniques. Proteomics approaches have been used to determine plants’ proteome responses under drought stress. For instance, Liu et al. determined the protein changes of mulberry in response to drought stress using tandem mass tags (TMT)-label LCMS/MS technique [10]. The authors found that proteins involved in photosynthesis, energy and sugar metabolisms, antioxidant production, hormones, and cell homeostasis were abundantly changed under drought conditions. Using the same technique, Xiao et al. identified 123 differentially changed proteins between 30-d drought-stressed cotton fine roots and control [11]. The number of proteins was increased to 1273 when cotton was exposed to 45-d drought treatment. Goche et al. reported that 237 and 187 root proteins were significantly altered in drought-susceptible and drought-tolerant sorghum varieties, respectively [12]. Other proteomics drought studies have also been reported on chickpea [13], grapevine [14], wheat [15], and banana [16].

*Pandanus amaryllifolius* is a member of the screw pine family Pandanaceae. This plant is commonly known as fragrant screw pine, pandan (Malaysia and Indonesia), pandan mabango (Philippines), and toei hom (Thailand). *P. amaryllifolius* is widely cultivated in Southeast Asia, such as in Thailand, Indonesia, and Malaysia. The pandan plants grow in clumps with leaf size reaching 40–80 cm tall and a width of about 4.5 cm. Its leaves are dark green with sharp spines on the margins and are commonly used as food flavoring, natural colorants, and herbal medicine. Besides its aromatic value, *P. amaryllifolius* leaves have also been found to contain phenolic compounds that possess health benefits. For instance, Ghasemzadeh and Jaafar reported that gallic acid and cinnamic acid isolated from *P. amaryllifolius* could inhibit 78% of breast cancer MCF-7 cell lines [17]. In addition, metabolic syndromes, such as weight gain, abdominal adipose tissue deposition, and blood pressure, can be reduced after treatment with the leaf extract of *P. amaryllifolius* [18].

*Pandanus* spp. is a moderate drought-tolerant plant [19]. For example, *P. tectorius* can survive in drought conditions for more than 6 months, whereas *P. odaritissimus* can survive under an area with rainfall of less than 2000 mm annually [20]. However, there is insufficient information on the extent of the drought tolerance of *P. amaryllifolius* despite its medicinal value. Understanding the plant’s strategy to adapt and survive under drought stress may refine our understanding of drought stress responses in plants and help to develop drought-tolerant crops. In this study, we examined the growth morphology, antioxidant enzymatic activities, and protein changes between drought-stressed, well-watered, and water-recovered *P. amaryllifolius* plants.

## 2. Results

### 2.1. Morphological Changes of Drought-Stressed Pandanus amaryllifolius

**Figure 1:**
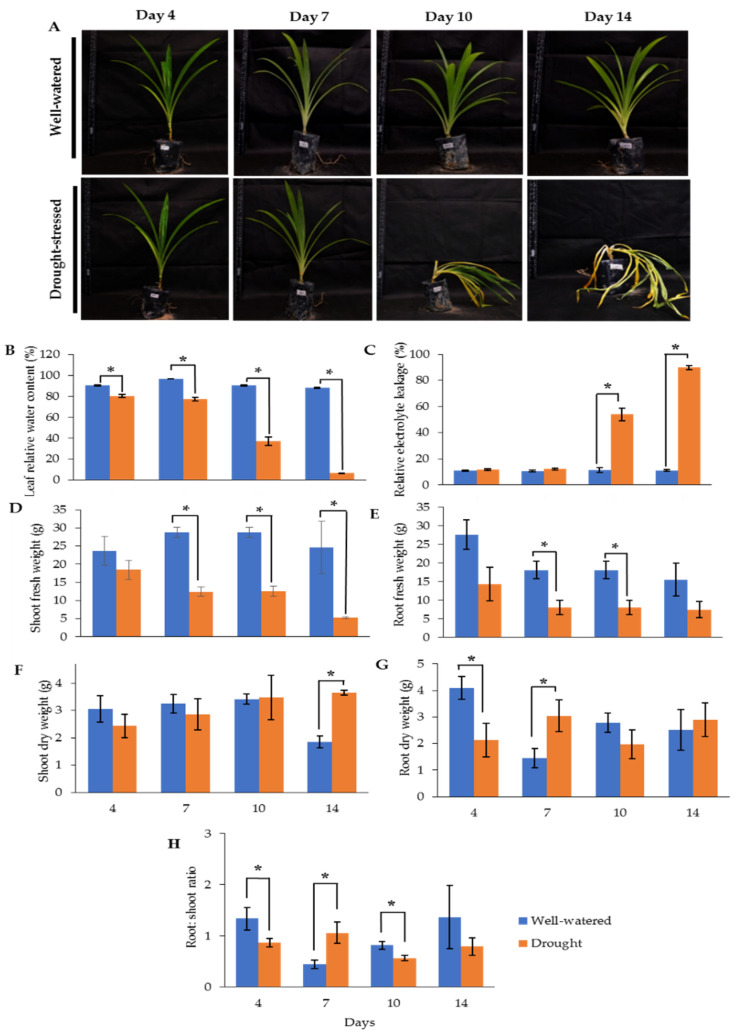
Responses of drought-stressed and well-watered *Pandanus amaryllifolius*. *P. amaryllifolius* plants were subjected to drought stress by withholding water for 4, 7, 10, and 14 days. Well-watered *P. amaryllifolius* was served as control. (**A**) Photographs of *Pandanus* plants were taken at 4, 7, 10, and 14 days. (**B**) The percentage of LRWC of *P. amaryllifolius* leaves at different time points. (**C**) The percentage of REL for each sample at different harvest points. (**D**) Shoot fresh weight of *Pandanus*. (**E**) Root fresh weight of *Pandanus*. (**F**) Shoot dry weight of *Pandanus*. (**G**) Root fresh weight of *Pandanus*. (**H**) Root-to-shoot ratio of *Pandanus* dry weight. Means labeled with asterisk were significantly different based on the Student’s *t*-test when its *p*-value < 0.01.

**Figure 2:**
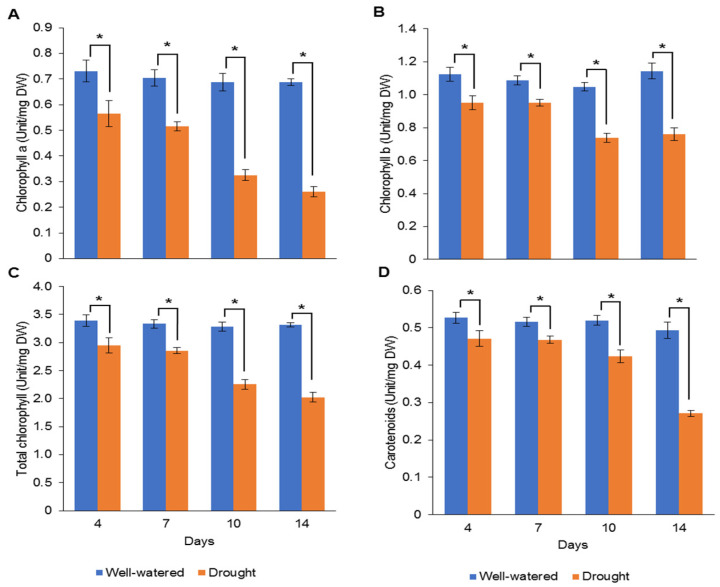
Pigment contents of leaf samples for well-watered and drought-stressed *Pandanus amaryllifolius* at different timepoints. The concentrations of (**A**) chlorophyll a (U mg^−^^1^ DW), (**B**) chlorophyll b (U mg^−^^1^ DW), (**C**) total chlorophyll (U mg^−^^1^ DW), and (**D**) carotenoid (U mg^−^^1^ DW). Means labeled with asterisk (*) were significantly different based on the Student’s *t*-test when its *p*-value < 0.01.

**Figure 3:**
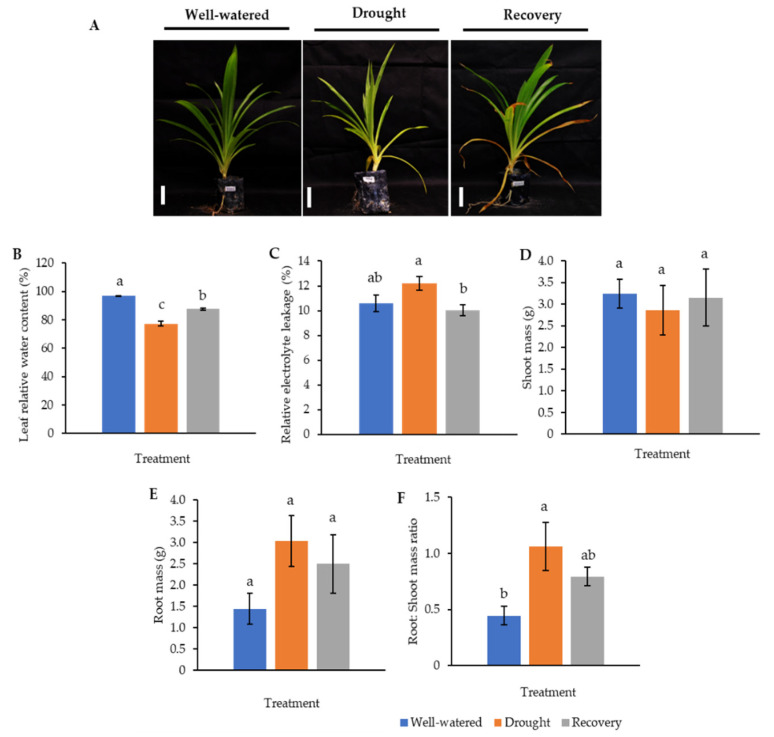
Comparison of well-watered, drought-stressed, and water-recovered *Pandanus amaryllifolius* plants. (**A**) *Pandanus* plants were subjected to drought stress by withholding water for 7 days, whereas the water-recovered plants were rewatered after a 7-day drought treatment and rewatered for 7 days. Well-watered *P. amaryllifolius* served as control. The line bar indicates the scale of the plant = 10 cm. (**B**) The percentage of leaf relative water content of *P. amaryllifolius* leaves. (**C**) The percentage of relative electrolyte leakage for each sample. (**D**) Shoot mass of *P. amaryllifolius*. (**E**) Root mass of *P. amaryllifolius*. (**F**) Root-to-shoot mass ratio of *P. amaryllifolius*. Means labeled with alphabet were significantly different based on the ANOVA followed by post hoc when its *p*-value < 0.05.

Our earlier preliminary experiment which exposed Pandanus plants to drought stress conditions for up to one month revealed that the plants could not survive after 14 days (data not shown). Hence, in the current study, we determined the early- to mid-drought response of Pandanus plants. At the morphological level, the changes of Pandanus plants in response to drought stress at several time intervals were determined (Figure 1A). The percentage of leaf relative water content (LRWC) for drought-stressed samples was significantly reduced after 4 days of drought treatment, whereas the percentage of relative electrolyte leakage (REL) for drought-stressed samples was significantly increased after 10 days of drought treatment (Figure 1B,C). The fresh and dry weights of shoots and roots in well-watered plants were generally higher than drought-stressed Pandanus (Figure 1D–G). Surprisingly, the root dry weight of the 7-day drought-stressed plants was significantly higher compared to well-watered plants (Figure 1G). Similarly, the root-to-shoot ratio of the 7-day drought-stress samples was higher than well-watered plants (Figure 1H). However, the chlorophyll a, chlorophyll b, total chlorophyll and carotenoid contents of the drought-stress samples were significantly reduced compared to well-watered plants (Figure 2A–D). After rewatering, only 7-day drought-stressed Pandanus plants were able to recover (Figure 3A). Hence, day 7 of drought treatment was selected for the subsequent experiments.

To understand the drought-responsive mechanism of Pandanus plants, a new set of experiments comprising well-watered, 7-day drought-stressed, as well as 7-day drought-stressed and rewatered plants was conducted since 10-day drought-stressed samples were unable to recover after rewatering. These samples were also subjected to morphological, biochemical, and proteomics analysis.

**Figure 4:**
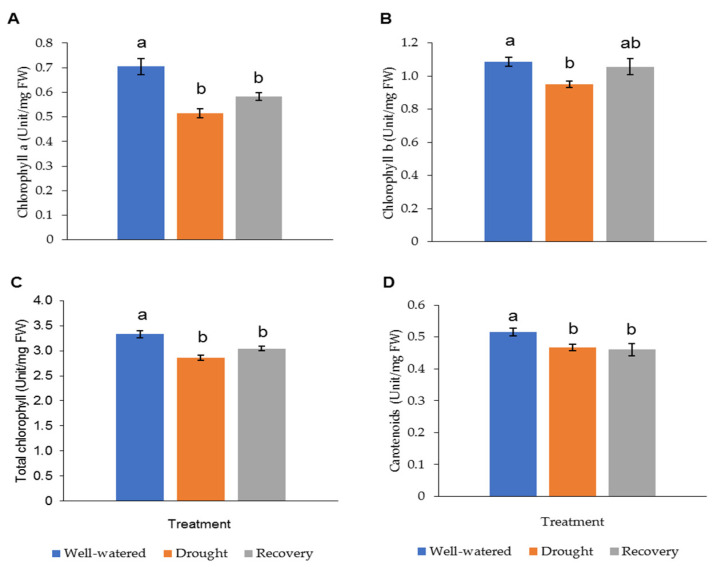
Pigment content of leaf samples for well-watered, drought-stressed, and recovered *Pandanus amaryllifolius*. The concentrations of (**A**) chlorophyll a (U mg^−1^ DW), (**B**) chlorophyll b (U mg^−1^ DW), (**C**) total chlorophyll (U mg^−1^ DW), and (**D**) carotenoids (U mg^−1^ DW). Means labeled with alphabet were significantly different based on the ANOVA followed by post hoc when its *p*-value < 0.05.

The LRWC in Pandanus plants was decreased with decreasing soil moisture content (Figure 3 and Figure S1). In particular, the drought-stressed Pandanus plants showed a 20% reduction compared to well-watered plants but recorded comparable LRWC with well-watered plants after rewatering (Figure 3B). The REL of the drought-stressed samples was higher than the well-watered and recovered plants (Figure 3C). The imposed drought stress did not affect the mass of both shoots and roots (Figure 3D,E). However, the root-to-shoot ratio in drought-stressed Pandanus was significantly higher than other treatments (Figure 3F). The pigment content of leaf samples in the drought-stressed Pandanus plants was significantly decreased (Figure 4A–D). It is worth mentioning that the leaf chlorophyll and carotenoid contents of the recovered plants were the same as drought-stressed plants, which could be related to insufficient recovery time (Figure 4A–D).

The Pandanus leaves showed slight wilting and clamping after 7 days of drought treatment but recovered after rewatering. However, there was a browning effect observed on the tips of mature leaves (Figure S2A). The yellow-green leaf color pigment pixel percentage in drought-stressed and recovered plants was slightly higher than well-watered plants (Figure S2B).

### 2.2. Changes of Proline and Malondialdehyde Contents in Pandanus Plants under Drought Stress

**Figure 5:**
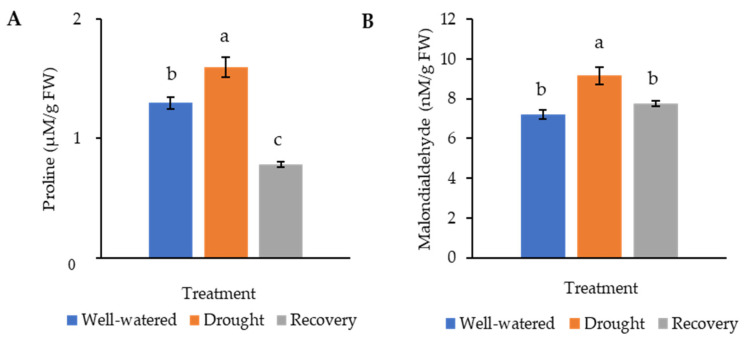
Osmolyte and lipid peroxidation changes of the drought-stressed and well-watered *Pandanus amaryllifolius*. (**A**) Proline content of *P. amaryllifolius* leaves as quantified in µM g^−1^ fresh weight (FW). (**B**) Malondialdehyde (MDA) content of *P. amaryllifolius* leaves as quantified in µM g^−1^ FW. Means labeled with alphabet were significantly different based on the ANOVA followed by post hoc when its *p*-value < 0.05.

Proline, being an osmoprotectant, is involved in protecting plants from harmful effects caused by environmental stresses. Malondialdehyde (MDA), which results from the breakdown of polyunsaturated fatty acids, is the product of membrane lipid peroxidation. Both proline and MDA serve as an indicator of stress tolerance. In this study, the proline content of the drought-stressed Pandanus plants (1.6 μM g^−^^1^ FW) was significantly higher than well-watered (1.3 μM g^−^^1^ FW) and recovered plants (0.8 μM g^−^^1^ FW) (Figure 5A). Similarly, the MDA content of the drought-stressed Pandanus plants was the highest (9.2 nM g^−^^1^ FW) (Figure 5B). The well-watered and recovered samples recorded the same MDA content (7.7 nM g^−^^1^ FW).

### 2.3. Antioxidant Enzyme Changes in Pandanus Plants in Response to Drought Stress

**Figure 6:**
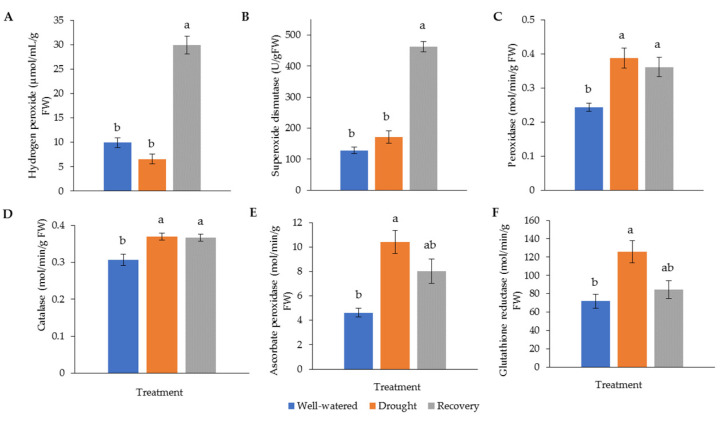
Activity of (**A**) hydrogen peroxide (H_2_O_2_) and antioxidant enzymes, (**B**) superoxide dismutase (SOD), (**C**) catalase (CAT), (**D**) peroxidase (POD), (**E**) ascorbate peroxidase (APX), and (**F**) glutathione reductase (GR) in *Pandanus amaryllifolius* leaves in response to drought stress and water recovery. Absorbance was measured through a spectrophotometer. H_2_O_2_ accumulation is shown in μM min^−1^ g^−1^ fresh weight (FW), whereas SOD is shown as U g^−1^ FW based on NBT coloration and inhibition. CAT, POD, APX, and GR are shown in M min^−1^ g^−1^ FW. Means labeled with alphabet were significantly different based on the ANOVA followed by post hoc when its *p*-value < 0.05.

Drought stress generally increases the activity of antioxidant enzymes. The highest hydrogen peroxide (H_2_O_2_) (30.0 μM min^−^^1^ g^−^^1^) and superoxide dismutase (SOD) (462.1 g^−^^1^ FW) levels were recorded in water-recovered plants (Figure 6A,B). Catalase (CAT) and peroxidase (POD) for both well-watered and recovered plants showed significantly higher activity than for drought-stressed plants (Figure 6C,D). The highest activity of ascorbate peroxidase (APX) (10.4 M min^−^^1^ g^−^^1^) and glutathione reductase (GR) (126.0 M min^−^^1^ g^−^^1^) was found in drought-stressed samples (Figure 6E,F).

### 2.4. Protein Changes in Well-Watered, Drought-Stressed, and Recovered Pandanus Plants

To identify the protein changes of Pandanus plants under drought stress, total protein from well-watered, drought, and water-recovered plants were extracted for nano-LC-MS/MS analysis. Of the 1415 identified proteins, 74 proteins were found to be significantly altered (Table 1). These proteins were visualized with hierarchical clustering (Figures S3A and S4) and clustered into four groups based on the log ratio expression between treatments (Figure S3B). Cluster 1 showed that 12 proteins in the well-watered samples were decreased in abundance when exposed to drought stress (Figure S3B). In contrast, cluster 4 indicated that nine proteins in the recovered samples were increased in abundance when compared to drought-stressed samples (Figure S3B).

**Figure 7:**
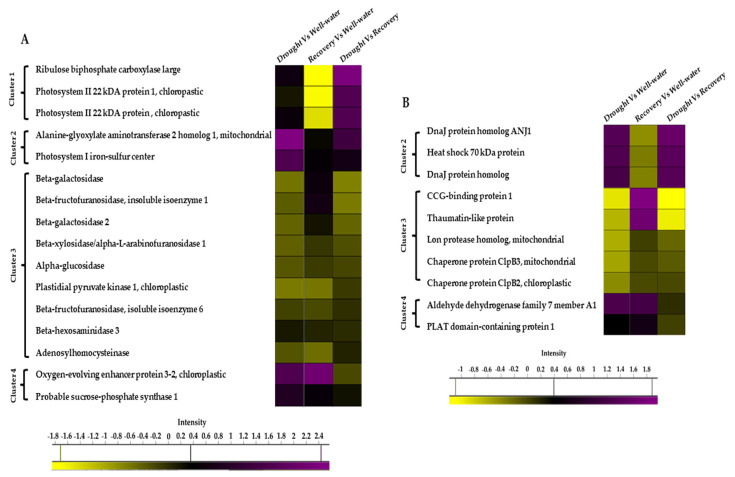
Heat map of the differentially changed protein classes identified between well-watered, drought-stressed, and water-recovered *Pandanus amaryllifolius*. (**A**) Carbon-related proteins identified between treatments. (**B**) Stressed-related proteins identified between treatments. The intensity scale indicates the range of upregulation (purple) or downregulation (yellow) of proteins between treatments.

**Figure 8:**
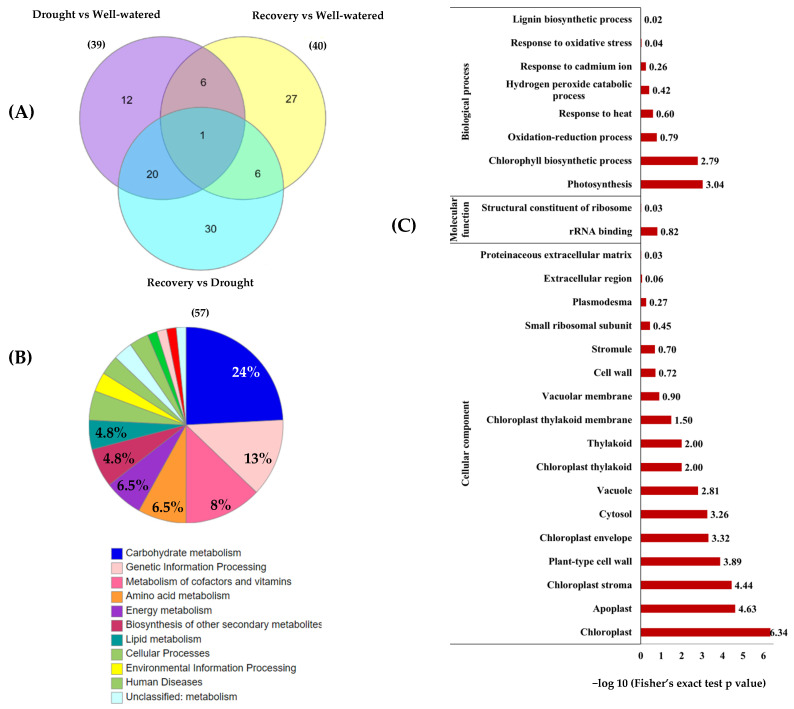
Functional categorization and enrichment of differentially changed proteins between well-watered, drought, and recovery samples. (**A**) The Venn diagram represents the comparison of differentially abundant proteins identified in the leaves of *Pandanus* plants treated with drought stress, well-watered, and recovery; (**B**) KEGG enrichment of differentially changed proteins based on functional category; and (**C**) gene ontology enrichment based on KEGG pathway according to biological processes, molecular functions, and cellular components.

Among the 74 identified proteins, carbohydrate- and stress-related proteins were the largest differentially changed protein group in this study (Figure 7A,B). Of these, 39 unique proteins were found in Comparison 1 (Drought vs. Well-watered), 40 proteins in Comparison 2 (Recovery vs. Well-watered), and 57 proteins in Comparison 3 (Recovery vs. Drought) (Figure 8A).

To classify the function of the 74 differentially changed proteins, KEGG pathway enrichment analysis was performed (Figure 8). The results showed that 15 of differentially changed proteins were involved in carbohydrate metabolism and another 8 proteins are involved in genetic information processing and cofactors and vitamin metabolism (Figure 8B). Based on the gene ontology (GO) functional classification, most of the differentially changed proteins were involved in photosynthesis processes and stress responses (Figure 8C). 

## 3. Discussion

### 3.1. Drought Stress Affected the Growth and Antioxidant Enzyme Activities of Pandanus Plants

Drought stress significantly affected the growth of *Pandanus* plants, such as LRWC, root-to-shoot ratio, shoot and root biomass, REL, and chlorophyll contents, leading to an accumulation of ROS that can damage cell membranes. A decline in leaf water content could reduce the cell turgor pressure and affect the strength and mechanical structure of the plants [21]. This was observed in the leaf morphological responses in drought-stressed *Pandanus* plants. Moreover, the percentage of REL was significantly increased in drought-stressed *P. amaryllifolius*, suggesting that cell membrane integrity is likely to be affected due to oxidative stress. In contrast, the rewatered *Pandanus* showed reduced REL. In sugarcane, an increase of 62% REL was found in the drought-stressed samples compared to control [22] but reduced after rewatering [23]. Similarly, Oraee and Tefranifar found that drought treatment increased the REL in pansy plants, while the REL in the rewatered drought-stressed pansy was reduced [24].

It is commonly known that the photosynthetic activity of plants is affected by drought stress [25,26]. Hence, it was not surprising that *Pandanus* plants exposed to drought stress showed a reduction in chlorophyll and carotenoid contents. As chloroplast is the main production site of ROS, an increased accumulation of ROS may damage the photosynthetic machinery. However, it is worth noting that the recovered plants showed similar chlorophyll and carotenoid contents as drought-stressed plants, suggesting that the degradation of pigments was halted during the recovery period. Nevertheless, a longer duration of rewatering might be required to allow the plants to recuperate their pigment constituents. The concentration of chlorophyll b was higher than chlorophyll a, which gave a lower chlorophyll a:b ratio. The probable cause of this phenomenon could be due to the imparting of the light intensity in the controlled growth room, where plants adapt themselves by increasing the amount of chlorophyll b pigments to capture a broader range of light [27]. Similar findings were reported by Yang et al. [28], where soybean leaves produced lower chlorophyll a than b under low light intensity.

Drought stress generally leads to the accumulation of MDA and proline [29]. Our results clearly showed that drought stress increased MDA and proline contents in *P. amaryllifolius*. Conversely, rewatering reduced MDA and proline contents, indicating the capacity of *P. amaryllifolius* to maintain the membrane integrity and restore its functions. These findings agree with previous studies, where MDA and proline were elevated under drought stress in various plants [30,31]. MDA is an index of oxidative injury, whereby MDA production correlates with ROS scavenging ability [32]. Proline is an essential adaptive response to drought stress in plants. It acts as an ROS scavenger, osmoprotectant, redox balancer, detoxification activator, and source for nitrogen and carbon [33].

ROS, such as superoxide (O_2_^•‒^), free radical (^•^OH), and H_2_O_2_, is generated and accumulates in plants in response to drought stress [34]. Antioxidant enzyme system protects plants against abiotic stress via ROS scavenging mechanism. In this study, the activity of antioxidant enzymes, such as CAT, APX, POD, and GR, in drought-stressed plants was generally higher than in well-watered plants. Higher antioxidant activities have contributed to a low level of H_2_O_2_ in the stressed plant. While SOD constitutes the first line of defense against ROS, its activity in drought-stressed *Pandanus* plants was comparable to the well-watered plants. This suggests that the detoxification of ROS that comprises mainly H_2_O_2_ was apprehended by CAT, APX, POD, and GR. Taken together, the enhanced antioxidant enzyme system helps *Pandanus* plants to cope with accumulated ROS under drought conditions.

### 3.2. Stress and Defense Protein Abundance under Drought Stress

Plants respond and adapt to drought stress conditions by changing regulatory circuits in transcription and protein expression, reorganizing metabolic pathways, and physiological processes [16]. This is the first study to determine protein changes in response to drought stress in *Pandanus* using a TMT-labeled proteomics approach. Proteins involved in stress-responsive and carbohydrate metabolism were identified as the largest differentially changed protein group in this study, suggesting their important roles in the response to drought stress conditions.

In the present study, the abundance of heat shock proteins 70 (HSP70) and DnaJ homolog proteins in drought-stressed samples was increased compared to other treatments. HSPs are molecular chaperones that protect plants by maintaining proper folding and preventing the aggregation of proteins [35]. Proper protein folding and disaggregation are vital for cell survival under stress conditions. To date, several members of the *HSP70* gene family have been identified in various plant species. Of these, some HSP70 members have been reported to be involved in the drought stress response. For instance, HSP70 in peanut was increased in abundance in response to drought [36]. DnaJ homolog proteins are essential components in the chaperone machine and could be induced by cold [37], heat [38], and drought stresses [39,40]. Other studies showed that several HSPs, co-chaperone DnaJ, and Grp chaperonin 60b were differentially altered under drought conditions [41,42]. Emerging evidence indicates that the accumulation of HSPs is correlated with stress tolerance in plants, as these proteins are only induced upon stress treatment [43]. These studies have revealed the importance of HSP70 and DnaJ-like proteins in conferring abiotic stress tolerance in plants.

Polycystin, lipoxygenase, alpha-toxin, and triacylglycerol lipase (PLAT) domain proteins are induced by abscisic acid and are involved in the abiotic stress response. These proteins mediate the abscisic acid signaling pathway after binding to bZIP transcription factors AREB/ABFs. PLAT protein was found to be abundantly accumulated in this study. The amount of PLAT was also increased in salinity-stressed quinoa [44]. Hyun et al. investigated the function of the Arabidopsis PLAT, *AtPLAT1* in tobacco [45]. The authors found that expressing *AtPLAT1* in tobacco promoted growth and increased stress tolerance towards cold, drought, and salt. However, the role of PLAT proteins in the drought stress response is still lacking and needs further exploration.

### 3.3. Cell Membrane-Related Proteins Increased under Drought Stress

Networked (NET) actin-binding proteins increased in drought-stressed *Pandanus* plants compared to well-watered plants, probably due to the need of maintaining plant cell integrity. NET proteins associate with different membrane compartments in plant cells and facilitate membrane–actin interaction [46]. At the transcript level, Li et al. found that *NET1A* gene in cotton was induced by drought stress, and its expression was significantly different between control and drought treatments [47]. Although the role of NET proteins in the stress response is still unclear, it was proposed that mechanical, gravitational, and osmotic stresses could directly induce the accumulation of NET proteins [48].

Alpha-L-arabinofuranosidase (ASD) is a cell wall protein, catalyzing the hydrolysis of L-arabinofuranosidic bonds in L-arabinose-containing hemicelluloses [49]. In this study, ASD1 protein was increased when exposed to drought stress. The role of ASD in drought stress has been shown in maize [50] and maritime pine [51].

### 3.4. Carbohydrate Metabolism-Related Protein Abundance under Drought Stress

Proteins involved in carbohydrate metabolism were increased in abundance, most probably due to energy demand caused by drought stress. One of the prominent proteins in this category is sucrose phosphate synthase (SPS). When plants experience drought stress, SPS catalyzes the conversion of uridine diphosphate-glucose and fructose 6-phosphate into sucrose. Nemati et al. found that SPS changed in abundance after being exposed to drought stress in wheat seedlings [52]. Other studies showed that drought treatment markedly increased SPS activity [53,54]. The high abundance of SPS shown by other studies in different drought-stressed plant species might be due to the need of cells for extra energy to cope with stress.

The overall results show that the response of Pandanus leaves to drought stress involves various biological processes, including an increase in abundance of most proteins related to stress and defense, cell membrane, and carbohydrate metabolisms. These protein changes could contribute to plant morpho-physiological changes to tolerate the water depriving conditions. Our findings suggest that, besides stress and defense proteins, the modulation of cell membrane- and carbohydrate metabolism-related proteins could be a valuable strategy to sustain plant metabolic processes in response to drought stress. Preserving cell membrane stability could help plants maintain a normal physiological metabolism under drought stress. On the other hand, enhanced carbohydrate metabolism could assist drought-stressed plants in maintaining normal growth by increasing energy production. Therefore, these proteins could be used as protein markers to identify drought tolerance in plants and may further assist plant biologists in selecting desired genes to develop new drought-tolerant varieties. We envisage that our proteomics data sets provide a foundation for further research that will strengthen our understanding of how crop plants respond to environmental stresses for better survival.

## 4. Materials and Methods

### 4.1. Plant Material and Drought Treatment

Three-month-old disease-free *P. amaryllifolius* purchased from the I Green Nursery Sdn. Bhd., Muar, Johor, Malaysia, were transferred to polybags (20 × 11 cm^2^) containing 600 g clay silt loam soil. The plants were acclimatized in a growth room at Universiti Malaya, Malaysia, and maintained at 28 ± 2 °C with a light intensity of 1500 lux under a 12:12 h photoperiod cycle and 80 ± 5% relative humidity for 2 weeks. All plants received 20 mL water once a day and foliar fertilizers once per week prior to treatment. The drought treatment was conducted by withholding water for 4, 7, 10, and 14 days, and rewatered for 7 days after drought treatment. Well-watered plants were watered daily throughout the experimental period. Based on the morphological results, an appropriate time point was then selected for biochemical and proteomics analysis. The soil moisture content in all pots was monitored every day using a soil moisture meter (HH2 moisture meter, Delta-T, Cambridge, UK). The experiments were carried out in a completely randomized block design with 8 biological replicates for each treatment.

### 4.2. Determination of Leaf Relative Water Content

Leaf relative water content (LRWC) was measured according to Turner [55]. Briefly, the harvested leaf samples were cleaned with 70% ethanol, cut into small pieces (2 cm^2^), and weighed for their fresh weight (FW) before being transferred to a Petri dish. The leaf samples were submerged in 20 mL of distilled water for 6 h at room temperature. The saturated weight (SW) of the samples was measured before being oven-dried for 2 days. The dried samples were then measured for their dry weight (DW). LRWC was calculated using the following formula:

LRWC (%) = [(FW − DW)/(SW − DW)] × 100(1)

### 4.3. Measurement of Relative Electrolyte Leakage

The relative electrolyte leakage (REL) was measured according to Quan et al. [56]. About 100 mg leaves were immerged in 10 mL deionized water and incubated at room temperature for 6 h under shaking condition of 150 rpm. After measuring initial electrical conductivity (C_i_) using a conductivity meter (Cyberscan CON 11, Eutech Instrument, Thermofisher, Singapore), the leaf samples were boiled for 20 min and measured for the conductivity of lysed cells (C_max_). REL was calculated as:

REL (%) = C_i_/C_max_ × 100 (2)

### 4.4. Measurement of Plant Weight

The fresh and dry weights of the leaves and roots were measured separately. The fresh weight of the samples was measured immediately after harvesting. The samples were then stored in a paper bag prior to drying in an oven for 7 days or until a constant weight was achieved to measure their dry weight. The dry weight of the roots and leaves was used to calculate the root-to-shoot ratio.

### 4.5. Automated Colorimetric Assay

Leaf color was evaluated by harvesting 6 leaf tips (10–12 cm in length), wiped with 70% ethanol, and arranged on a white background. Leaves were photographed by using Nikon D5100 (Nikon Corp, Tokyo, Japan), processed by Image J software [57], and analyzed by automated colorimetric assay [58]. The pixels were grouped into four categories, namely green, green-yellow, yellow, and brown.

### 4.6. Measurement of Chlorophyll Content

The chlorophyll content was determined according to [59] with minor modifications. The leaf powder was subjected to freeze dryer overnight to remove all the water content in the cells before extraction of pigments. About 0.1 g freeze dried leaf powder was mixed well with 2 mL 80% (*v*/*v*) acetone in a 2 mL microcentrifuge tube. The mixture was incubated in the dark for 20 min before centrifuged at 10,000× *g* rpm for 15 min. About 100 µL supernatant was mixed with 900 µL 80% (*v*/*v*) acetone. The absorbance of the mixture was measured by a spectrophotometer at 470, 647, and 663 nm. The chlorophyll and carotenoid contents were calculated according to the formula below:

Chlorophyll a, C_a_ = 12.25A_663_
**−** 2.79A_647_ (3)

Chlorophyll b, C_b_ = 21.50A_647_
**−** 5.10A_663_ (4)

Total chlorophyll, C_a+b_ = 7.15A_663_ + 18.71A_647_ (5)

Total carotenoids, C_x+c_ = (1000A_470_
**−** 1.82C_a_
**−** 85.02C_b_) ÷ 198 (6)

### 4.7. Determination of Malondialdehyde Content

Malondialdehyde (MDA) content was measured as described by Heath and Packer [60]. Fresh leaf samples (100 mg) were homogenized in liquid nitrogen, then extracted with 1.5 mL 0.1% (*w*/*v*) trichloroacetic acid (TCA), and centrifuged at 13,000× *g* rpm for 10 min at 4 °C. About 300 µL supernatant was added into a 1 mL reaction mixture containing 0.5% (*v*/*v*) thiobarbituric acid (TBA) in 20% (*w*/*v*) TCA. The mixture was heated at 95 °C for 30 min, cooled on ice, and centrifuged at 10,000× *g* rpm for 10 min. The supernatant was measured using a spectrophotometer at wavelengths 532 and 600 nm and calculated using the following formula:

MDA = [(A_532_ − A_600_) × VTr × 1000]/(Extinction coefficient MDA × 1 cm × Ve) ÷ g FW (7)

A_532_ − A_600_ = Absorbance of MDA-TBA.

VTr = Volume of reaction (mL).

Ve = Volume of enzyme extract (mL).

Extinction coefficient of this MDA-TBA abduct at 532 nm is 155 mM^−^^1^ cm^−^^1^.

FW = Fresh weight of the sample.

### 4.8. Measurement of Proline Content

Proline content was measured according to Bates et al. [61] with modifications. About 200 mg leaf samples were ground with 2 mL 70% (*v*/*v*) ethanol and the mixture was centrifuged at 13,000× *g* rpm for 20 min. The supernatant was added to a reaction mixture containing 500 µL glacial acetic acid:500 µL freshly prepared acid-ninhydrin reagent:500 µL sample extract or proline standards. The mixture was mixed and boiled at 100 °C in a heat block for 1 h. After placing on ice for 30 min, the mixture was extracted with 1 mL toluene. The toluene phase was carefully collected into a test tube. The absorbance of the fraction was measured at 520 nm with toluene as blank. The proline standard curve was constructed at concentrations of 1, 5, 10, 25, 50, 100, and 200 µM. The proline content was calculated based on the following formula:

Proline (µMg^−^^1^ FW) = [(μg proline/mL × mL toluene) ÷ μg 115.5/μmole]/(g FW/5) (8)

### 4.9. Antioxidant Enzymatic Assays

About 200 mg leaf samples were mixed with 2 mL cold extraction buffer (100 mM phosphate buffer, pH 7.0, containing 0.1 mM disodium ethylenediaminetetraacetic acid, and 0.1 g polyvinylpyrrolidone). The mixture was centrifuged at 13,000× *g* rpm for 10 min at 4 °C. The supernatant was used for the subsequent antioxidant enzymatic assays. The enzyme activity for each assay was calculated using the following formula:

Enzyme activity (M min^−^^1^ g^−^^1^ FW) = (∆A× VTr)/(ε × ∆t × 1 cm × Ve × g FW) × 1000 (9)

∆A = Difference in absorbance.

VTr = Volume of reaction (mL).

Ve = Volume of enzyme extract (mL).

∆t = Difference in time of absorbance (min).

For CAT, ε(Hydrogen peroxide) = 36.0 mol^−^^1^ cm^−^^1^.

For APX, ε(Ascorbic acid) = 2.8 mmol^−^^1^ cm^−^^1^.

For POD, ε(Guaiacol) = 26.6 mol^−^^1^ cm^−^^1^.

For GR, ε(NADPH) = 6220 mol^−^^1^ cm^−^^1^**_._**

#### 4.9.1. Superoxide Dismutase

SOD activity was determined according to Dhindsa et al. [62]. Briefly, 3 mL of reaction mixture containing 50 mM phosphate buffer (pH 7.0), 9.9 mM L-methionine, 55 µM nitro blue tetrazolium (NBT), 0.025% (*v*/*v*) Triton X-100 (Sharlau, Mahmutbey, Turkey), 100 µL enzyme extract, and 4.8 µM riboflavin was prepared in a test tube and covered by aluminum foil. The riboflavin was added last to initiate the reaction. The mixture was shaken and incubated at 30 °C for 10 min under a white light source (35 W) placed at 20 cm height above the test tubes. The mixture was measured at 560 nm. The blank was prepared by replacing the sample with extraction buffer. The SOD activity was calculated based on the formula below:

SOD (Unit g^−1^ FW) = [(Blank- Sample) A_560nm_]/(Blank A_560nm_)] × (Volume reaction)/(Volume enzyme) × 100 × 1/50 ÷ 0.1 g FW (10)

#### 4.9.2. Catalase

CAT assay was performed as described by Aebi [63] with modifications. The 3 mL reaction mixture contained 50 mM phosphate buffer (pH 7.0), freshly prepared 8.33 mM H_2_O_2_, and 100 µL enzyme extract. The enzyme extract was added last to initiate the reaction. The CAT activity was measured at 240 nm.

#### 4.9.3. Ascorbate Peroxidase

APX activity was measured according to Chen and Asada [64]. The 1 mL reaction mixture contained 50 mM phosphate buffer (pH 7.0), 200 µL enzyme extract, 0.5 mM ascorbic acid, and 0.42 mM H_2_O_2_. H_2_O_2_ was added last to initiate the reaction. The APX activity was measured at 290 nm wavelength.

#### 4.9.4. Peroxidase

POD assay was carried out according to Maehly and Chance [65] with modifications. The 1 mL reaction mixture contained 100 mM phosphate buffer (pH 7.0), 0.5 mM guaiacol, 0.0833 mM H_2_O_2_, and 100 µL enzyme extract. H_2_O_2_ was added last to initiate the reaction. The POD activity was measured at 470 nm wavelength.

#### 4.9.5. Glutathione Reductase

GR activity was determined as described by Mannervik [66]. The 1 mL reaction mixture contained 500 µL assay buffer (0.2 M potassium phosphate buffer, pH 7.0, 0.2 mM EDTA, 50 µL 20 mM freshly prepared oxidized glutathione, 50 µL 2 mM NADPH solution, and 300 µL enzyme extract) and distilled water. The decrease in absorbance at 340 nm was monitored for 1 min.

#### 4.9.6. Hydrogen Peroxide

The H_2_O_2_ level in the leaf samples was determined according to Velikova et al. [67] with slight modifications. Briefly, 100 mg leaf powder was homogenized with 1.5 mL 0.1% (*w*/*v*) TCA in an ice bath. After centrifugation at 10,000× *g* rpm for 15 min, about 250 µL supernatant was mixed with 1 mL reaction mixture containing 2.5 mM potassium phosphate buffer, pH 7.0, and 0.5 M potassium iodide. The H_2_O_2_ concentration was measured at 390 nm and calculated using a standard curve with concentrations of 2.5 to 100 µM.

### 4.10. Protein Extraction and Quantification

Total protein from five biological replicates from each treatment was extracted using TCA/acetone precipitation with phenol method [68] with slight modifications. The leaf samples (200 mg) were extracted using 4 mL cold 10% (*w*/*v*) TCA/acetone containing 10 mM dithiothreitol (DTT) and 0.001% (*v*/*v*) protease inhibitor. The homogenate was centrifuged at 15,000× *g* rpm for 5 min at 4 °C. The pellet was resuspended with 1.5 mL 10% (*w*/*v*) TCA/acetone before centrifuged at 15,000× *g* rpm for 5 min at 4 °C. The pellet was then washed with 80% ice cold acetone containing 10 mM DTT. After being centrifuged at 15,000× *g* rpm for 5 min at 4 °C, the pellet was resuspended with 1.5 mL sodium dodecyl sulfate (SDS) extraction buffer (1% (*w*/*v*) SDS, 0.15 M Tris-HCl, pH 8.8, 0.1 M DTT, 1 mM EDTA, and 0.001% (*v*/*v*) protease inhibitor). The mixture was incubated at 65 °C for 1 h before being centrifuged at 15,000× *g* rpm for 10 min at room temperature and the supernatant was collected in a new tube. An equal volume of Tris-buffered phenol (pH 8.0) was then added to the supernatant. The phenol phase at the bottom layer was extracted and mixed with washing buffer (10 mM Tris-HCl (pH 8.0), 1 mM EDTA, and 0.7 M sucrose). After centrifugation, the upper layer was mixed with 1.5 mL 0.1 M ammonium acetate in methanol. The mixture was incubated at −20°C overnight and centrifuged at 15,000× *g* rpm for 10 min at 4 °C. The pellet was washed with 1.5 mL 0.1 M ammonium acetate in methanol, centrifuged at 15,000× *g* rpm for 5 min at 4 °C, and mixed with 1 mL 80% (*v*/*v*) ice cold acetone containing 20 mM DTT. After centrifugation, the pellet was vacuum-dried in a desiccator for 5 min and resuspended in either S-Traps lysis buffer (5% SDS, 100mM triethylammonium bicarbonate, TEAB, pH 7.55) (Protifi, Huntington, NY, USA) for LCMS/MS analysis or resuspension buffer (7 M urea, 2 M thiourea, 4% (*w*/*v*) 3-[(3-cholamidopropyl) dimethylammonio]-1-propanesulfonate (CHAPS), 30 mM Tris-HCl (pH 8.0), and 1% DTT) for protein content measurement. The protein content was determined using Bradford reagent [69], where bovine serum albumin was used as a protein standard.

### 4.11. Protein Preparation

The dried samples were sent to Australian Proteome Analysis Facility (APAF) for LC-MS/MS. About 140 μg total protein from each sample was resuspended in S-Trap lysis buffer and reduced by 10 mM DTT for 30 min at 56 °C and alkylated by 25 mM iodoacetamide (IAA) for 30 min at room temperature in the dark. The pH of the solution was adjusted by adding 12% aqueous phosphoric acid with 1:10 ratio for a final concentration of 1.2% and diluted by using S-Trap binding buffer (90% aqueous methanol containing 100 mM TEAB, pH 7.55). The S-Trap binding buffer was then added to the acidified lysis buffer. The mixture was transferred to a labeled S-Trap column before being centrifuged at 4000× *g*. S-Trap binding buffer was used to wash the column twice and the protein retained on the column was digested with 125 μL trypsin solution (~10 μg trypsin (Pierce, Thermo Scientific, Waltham, MA, USA) in 50 mM triethylammonium bicarbonate) at 47 °C for 3 h. Next, 50 mM triethylammonium bicarbonate was added to the column following centrifugation to elute off the peptides, while the remaining peptides were eluted from the column by sequential centrifugation with 0.2% aqueous formic acid followed by 50% aqueous acetonitrile (ACN) containing 0.2% formic acid. The peptides were vacuum centrifuged and resuspended in 110 μL 200 mM HEPES (pH 8.8). Each 10 μL sample was then pooled into a tube. The peptide concentration of the reconstituted solution was determined by using Pierce quantitative colorimetric peptide assay (Thermo Scientific).

### 4.12. Protein TMT-Labelling and Fractionation

All samples were labeled in a 10-plex TMT label (Thermo Scientific) and incubated at room temperature for 1 h with occasional vortexing. Next, 5% hydroxylamine was added to each sample, vortexed, and incubated at room temperature for 15 min to remove the excess TMT label. To ensure the number of peptides in each sample was equal, an equal amount of each TMT-labelled sample was mixed, vacuum dried, and resuspended in 2% ACN 0.1% formic acid to obtain the normalization factor. All TMT-labeled samples were pooled with a 1:1 ratio. The TMT-labeled peptides were vacuum dried, reconstituted in 0.1% trifluoroacetic acid and fractionated at three concentrations of 12.5%, 17.5%, and 50% ACN with 0.1% triethylamine from Pierce High pH Reversed-Phase Peptide Fractionation Kit (Thermo Scientific).

### 4.13. Liquid Chromatography-Tandem Mass Spectroscopy

One-dimensional data dependent acquisition (DDA) LC-MS/MS (Q-Exactive, Thermo Fisher) was performed by injecting each TMT-labeled peptide fraction onto the peptide trap, followed by a loading buffer (99.9% water and 0.1% formic acid). The peptides were eluted from the trap onto the nano-LC column and separated with linear gradient of 1% mobile phase A (99.9% water and 0.1% formic acid) to 30% over 110 min at 300 nL/min, followed by 85% B (99.9% ACN and 0.1% formic acid) for 8 min. The ionization source was set as positive ion mode, whereby 350–1850 m/z peptide precursors were scanned at 70,000 resolutions. The 10 most intense ions in the survey scan were subjected to the fragmentation by HCD using a normalized collision energy of 35 with precursor isolation width of 0.7 *m*/*z*. The precursors with charges of +2 to +4 were subjected to MS/MS analysis under the parameters: minimum signal of 6200 ions was required for MS2 triggering, an AGC target value of 2 × 10^5^ for MS2 and maximum injection time of 250 ms with 70,000 MS/MS scan resolution, and 90 s of dynamic exclusion.

### 4.14. Proteomics Data Analysis

The raw data files generated were searched against Viridiplantae protein sequences downloaded from UniProt (reviewed, 40,400 proteins sequences; 210312_uniprot-taxonomy_viridiplantae+reviewed.fasta) (accessed on March 2021) using Proteome Discoverer 2.1.0.81 (Thermo Scientific) and the Sequest HT search algorithm, allowing up to two missed cleavages per peptide and 20 ppm precursor mass tolerance. Carbonylation of cysteine was used as a fixed modification, whereas oxidation of methionine, deaminated (N, Q), Glu-> PyroGlu, Gln-> PyroGlu, N-Terminus acetylation, TMT6Plex (K), and TMT6Plex (N-Term) were used as variable modifications. Only high confidence peptides were selected using a percolator algorithm for the peptide-spectrum match (PSM) in database searches with false discovery rate (FDR) threshold set at 0.01 for protein identification. The identified proteins were filtered based on their abundance ratio of comparisons; Comparison 1 (Drought vs. Well-watered), Comparison 2 (Recovery vs. Well-watered), and Comparison 3 (Recovery vs. Drought), following *p*-value of less than 0.05 (Student’s *t*-test) and in the range of less than 0.5-fold change and more than 1.2-fold change of abundance.

### 4.15. Functional Classification of Proteins

The significantly differentially changed proteins were visualized by hierarchical clustering using Perseus software (version 1.6.0.7) [70]. These differentially changed proteins were then subjected to Kyoto Encyclopedia of Genes and Genomes (KEGG) (https://www.kegg.jp/) (accessed on 17 October 2021) for protein functional categorization, while enrichment pathway analysis was performed using DAVID functional annotation tools with *Arabidopsis thaliana* as background comparison.

### 4.16. Statistical Analysis

The morphological and antioxidant enzyme data at different time points were analyzed by Student’s *t*-test, whereby the analyzed data was considered to be statistically significant when its *p*-value < 0.01. The comparisons between well-watered, drought, and recovery samples were analyzed using analysis of variance (ANOVA) followed by a post hoc Tukey range using SPSS Statistics software (version 23.0; IBM) with *p*-value < 0.05 considered to be statistically significant.

## 5. Conclusions

The imposed drought stress affected the growth and development of *P. amaryllifolius*. The drought-stressed plants showed decreased LRWC and chlorophyll contents while REL, MDA, and proline contents were increased. The accumulation of ROS in drought-stressed plants enhanced their antioxidant enzyme activities. The drought-responsive mechanisms of *P. amaryllifolius* were determined at the proteome level. The majority of proteins differing between drought-stressed and well-watered plants were classified as heat shock proteins and photorespiration-related proteins, while proteins involved in carbon metabolism, chlorophyll biosynthesis, and antioxidants were significantly altered in water-recovered plants. Further research should be performed in field settings to better understand the drought-responsive mechanism in *P. amaryllifolius*.

## Figures and Tables

**Table 1:** List of abundantly altered protein profiles between well-watered, drought-stressed, and recovered *Pandanus amaryllifolius*.

Accession	Protein	Biological Process	Function	Cluster ^a^
F1SWA0	Zerumbone synthase	Protein synthesis	Oxidoreductase	1
P49043	Vacuolar-processing enzyme	Cysteine-type endopeptidase	Hydrolase	1
P48711	Ribulose bisphosphate carboxylase large chain	Photorespiration	Magnesium ion binding	1
Q9FLN4	50S ribosomal protein L27, chloroplastic	Ribonucleoprotein	mRNA binding	1
A0A357	30S ribosomal protein S18, chloroplastic	Ribonucleoprotein	rRNA binding	1
A1E9N5	30S ribosomal protein S7, chloroplastic	Ribonucleoprotein	rRNA binding	1
O23760	Caffeic acid 3-O-methyltransferase	Lignin biosynthesis	Methyltransferase	1
B2LMP1	30S ribosomal protein S15, chloroplastic	Ribonucleoprotein	Structural constituent of ribosome	1
A2WXD9	Photosystem II 22 kDa protein 1, chloroplastic	Photosynthesis	Non-photochemical quenching	1
Q9XF91	Photosystem II 22 kDa protein, chloroplastic	Photosynthesis	Non-photochemical quenching	1
Q32RY4	30S ribosomal protein S4, chloroplastic	Ribonucleoprotein	rRNA binding	1
O24461	Ras-related protein Rab7	Protein transport	GTPase activity	1
O22925	Vacuolar-sorting receptor 2	Protein transport	Calcium ion binding	2
Q940M2	Alanine-glyoxylate aminotransferase 2 homolog 1, mitochondrial	Photorespiration	Aminotransferase	2
Q9LUI2	Protein NETWORKED 1A	Cytoskeleton	Actin binding protein	2
P43644	DnaJ protein homolog ANJ1	Stress response	Chaperone	2
P11143	Heat shock 70 kDa protein	Stress response	Chaperone	2
A4QLY6	Photosystem I iron-sulfur center	Photosynthesis (ET)	Oxidoreductase	2
Q05737	GTP-binding protein YPTM2	Protein transport	GTPase activity	2
Q04960	DnaJ protein homolog	Stress response	Chaperone	2
Q9XIM0	CCG-binding protein 1	Cellular response to hypoxia	Mediator complex binding	3
P81370	Thaumatin-like protein	Plant defence	Pathogenesis	3
Q6DBP4	Pectin acetylesterase 8	Cell wall biogenesis/degradation	Hydrolase	3
Q9FLC0	Peroxidase 52	Hydrogen peroxide	Oxidoreductase	3
Q96520	Peroxidase 12	Hydrogen peroxide	Oxidoreductase	3
P48980	Beta-galactosidase	Carbohydrate metabolism	Glycosidase	3
Q01289	Protochlorophyllide reductase, chloroplastic	Chlorophyll biosynthesis	Oxidoreductase	3
P26792	Beta-fructofuranosidase, insoluble isoenzyme 1	Carbohydrate metabolism	Glycosidase	3
Q0DM51	DEAD-box ATP-dependent RNA helicase 3, chloroplastic	Ribosome biogenesis	Hydrolase	3
F6H7K5	Thiamine thiazole synthase 2, chloroplastic	Thiamine biosynthesis	Transferase	3
Q9LN49	3-ketoacyl-CoA synthase 4	Acyltransferase	Fatty acid biosynthesis	3
Q75LR2	Phospho-2-dehydro-3-deoxyheptonate aldolase 1, chloroplastic	Amino acid biosynthesis	Transferase	3
O82627	Granule-bound starch synthase 1, chloroplastic/amyloplastic	Starch biosynthesis	Glycosyltransferase	3
Q8W0A1	Beta-galactosidase 2	Carbohydrate metabolism	Glycosidase	3
O23787	Thiamine thiazole synthase, chloroplastic	Thiamine biosynthesis	Transferase	3
Q9ZQ94	UDP-glycosyltransferase 73C5	Brassinosteroid metabolism	Glycosyltransferase	3
O80731	Pectin acetylesterase 3	Cell wall biogenesis/degradation	Hydrolase	3
Q9C992	3-ketoacyl-CoA synthase 7	Acyltransferase	Fatty acid biosynthesis	3
O22436	Magnesium-chelatase subunit ChlI, chloroplastic	Chlorophyll biosynthesis	Ligase	3
Q84P54	Gamma aminobutyrate transaminase 1, mitochondrial	Biotin biosynthesis	Aminotransferase	3
A5JTQ2	Beta-xylosidase/alpha-L-arabinofuranosidase 1 (Fragment)	Carbohydrate metabolism	Glycosidase	3
Q42850	Protochlorophyllide reductase B, chloroplastic	Chlorophyll biosynthesis	Oxidoreductase	3
Q9SD46	Peroxidase 36	Hydrogen peroxide	Oxidoreductase	3
O04931	Alpha-glucosidase	Carbohydrate metabolism	Glycosidase	3
Q08937	29 kDa ribonucleoprotein B, chloroplastic	mRNA processing	Ribonucleoprotein	3
Q5ZE07	Multicopper oxidase LPR1 homolog 2	Phosphate homeostasis	Oxidoreductase	3
A4S6Y4	Lon protease homolog, mitochondrial	Oxidative stress	DNA binding	3
Q40147	Glutamate-1-semialdehyde 2,1-aminomutase, chloroplastic	Chlorophyll biosynthesis	Isomerase	3
Q9LIK0	Plastidial pyruvate kinase 1, chloroplastic	Glycolysis	Kinase	3
Q6STH5	Fe-S cluster assembly factor HCF101, chloroplastic	iron-sulphur cluster assembly	4Fe-4S cluster binding	3
Q0E3C8	Chaperone protein ClpB3, mitochondrial	Stress response	Chaperone	3
Q94LW3	Homeobox protein knotted-1-like 3	Mucilage biosynthesis	DNA binding	3
Q42600	Cytochrome P450 84A1	Phenylpropanoid biosynthesis	Monooxygenase	3
Q56UD0	Beta-fructofuranosidase, insoluble isoenzyme 6	Carbohydrate metabolism	Glycosidase	3
Q8L7S6	Beta-hexosaminidase 3	Carbohydrate metabolism	Glycosidase	3
Q39613	Peptidyl-prolyl cis-trans isomerase	Protein folding	Chaperone	3
Q9SJ20	Ribonucleoside-diphosphate reductase large subunit	DNA replication	Oxidoreductase	3
Q75GT3	Chaperone protein ClpB2, chloroplastic	Stress response	Chaperone	3
Q9ZUU4	RNA-binding protein CP29B, chloroplastic	mRNA processing	Ribonucleoprotein	3
Q9M591	Magnesium-protoporphyrin IX monomethyl ester [oxidative] cyclase, chloroplastic	Chlorophyll biosynthesis	Oxidoreductase	3
Q9CA67	Geranylgeranyl diphosphate reductase, chloroplastic	Chlorophyll biosynthesis	Oxidoreductase	3
P50246	Adenosylhomocysteinase	One-carbon metabolism	Hydrolase	3
Q6ZIV7	Hypersensitive-induced response protein 1	Potassium ion channel regulation	Histidine kinase binding	3
Q9SI75	Elongation factor G, chloroplastic	Protein biosynthesis	Elongation factor	3
P24846	4-hydroxy-tetrahydrodipicolinate synthase 1, chloroplastic	Amino acid biosynthesis	Allosteric enzyme	3
Q41932	Oxygen-evolving enhancer protein 3-2, chloroplastic	Photosynthesis (ET)	Calcium ion binding	4
P25795	Aldehyde dehydrogenase family 7 member A1	Stress response	Oxidoreductase	4
Q9AXH0	Catalase	Hydrogen peroxide	Oxidoreductase	4
O65660	PLAT domain-containing protein 1	Stress response	Catalase	4
A2YH64	Catalase isozyme B	Hydrogen peroxide	Oxidoreductase	4
Q0E4K1	Catalase isozyme A	Hydrogen peroxide	Oxidoreductase	4
O04932	Probable sucrose-phosphate synthase 1	Glycosyltransferase	Sucrose biosynthesis	4
Q570C8	3-ketoacyl-CoA thiolase 5, peroxisomal	Acyltransferase	Fatty acid biosynthesis	4
Q9SG80	Alpha-L-arabinofuranosidase 1	L-arabinose metabolic	Hydrolase	4

^a^ Clusters 1–4 show the differential accumulation of differentially changed proteins (log ratio expression) between treatments (Figure S3B). Cluster 1 represents the decreasing protein abundance from the well-watered to drought-stressed and recovery samples. Cluster 2 shows the increasing protein abundance pattern from the well-watered to drought-stressed but decreasing from drought-stressed to recovery samples. Cluster 3 represents the decreasing protein abundance when comparing well-watered to drought-stressed but increasing from the drought-stressed to recovery samples. Cluster 4 shows the increasing abundance of proteins from well-watered to recovery samples.

## Data Availability

The mass spectrometry proteomics data have been deposited to the ProteomeXchange Consortium via the PRIDE [71] partner repository with the dataset identifier PXD028784.

## Supplementary Materials

The following are available online at https://www.mdpi.com/article/10.3390/plants11020221/s1. Figure S1. Soil moisture content measured in well-watered, drought and recovery samples throughout the 14 days of experiment; Figure S2. Comparison of *Pandanus amaryllifolius* leaves under different treatment. (A) The leaves were arranged according to their position from the center (number 1) to the mature leaf (number 6) and the line bar = 5 cm. This layout was used for automated colorimetric assay (ACA); (B) The comparison of green and green-yellow pigments pixel percentage on each time point using ACA. Means labelled with alphabet was significantly different based on the ANOVA followed by post hoc when its *p*-value < 0.01; Figure S3. Hierarchal clustering of average protein abundance and clustering between treatment comparisons for well-watered, drought stressed, and recovered *Pandanus amaryllifolius*. (A) Heatmap of the significant protein abundance comparison between treatments according to its cluster group. The intensity scale indicates the range of upregulation (red) or downregulation (green) of proteins between treatments; (B) Significant differentially abundant proteins between treatments clustered into 4 groups according to the log ratio expression; Figure S4. Heatmap of the significant protein abundance comparison between treatments according to its cluster group. The intensity scale indicates the range of upregulation (purple) or downregulation (yellow) of proteins between treatments.

## References

[1] FAO (2018). The Impact of Disasters and Crises on Agriculture and Food Security: 2017.

[2] Foreign Agricultural Service USDA. https://www.fas.usda.gov/data/thailand-impact-drought-agriculture-2020.

[3] Zulkarami B., Razi I.M., Halimi M.S., Mondal M.A., Panhwar Q.A., Islam M.R. (2014). Effectiveness of different phytohormones on grain filling and yield of rice (*Oryza sativa* L.) under drought stress. J. Food Agri. Environ..

[4] Nalina M., Saroja S., Chakravarthi M., Rajkumar R., Radhakrishnan B., Chandrashekara K.N. (2021). Water deficit-induced oxidative stress and differential response in antioxidant enzymes of tolerant and susceptible tea cultivars under field condition. Acta. Physiol. Plant..

[5] Zhou J., Chen S., Shi W., David-Schwartz R., Li S., Yang F., Lin Z. (2021). Transcriptome profiling reveals the effects of drought tolerance in giant juncao. BMC Plant. Biol..

[6] Wang D., Chen Q., Chen W., Guo Q., Xia Y., Wang S., Jing D., Liang G. (2021). Physiological and transcription analyses reveal the regulatory mechanism of melatonin in inducing drought resistance in loquat (*Eriobotrya japonica* Lindl.) seedlings. Environ. Exp. Bot..

[7] Bankaji I., Sleimi N., Vives-Peris V., Gómez-Cadenas A., Pérez-Clemente R.M. (2019). Identification and expression of the cucurbita WRKY transcription factors in response to water deficit and salt stress. Sci. Hortic..

[8] Maurel C., Nacry P. (2020). Root architecture and hydraulics converge for acclimation to changing water availability. Nat. Plants.

[9] Santos J., Oliveira L.E., Coelho V.T., Lopes G., Souza T., Porto A.C., Lira J., Massote R., Rocha C., Gomes M.P. (2021). Performance of hevea brasiliensis under drought conditions on osmoregulation and antioxidant activity through evaluation of vacuolar invertase and reducing sugars. Plant. Sci. Today.

[10] Liu Y., Ji D., Turgeon R., Chen J., Lin T., Huang J., Luo J., Zhu Y., Zhang C., Lv Z. (2019). Physiological and proteomic responses of mulberry trees (*Morus alba* L.) to combined salt and drought stress. Int. J. Mol. Sci..

[11] Xiao S., Liu L., Zhang Y., Sun H., Zhang K., Bai Z., Dong H., Liu Y., Li C. (2020). Tandem mass tag-based (TMT) quantitative proteomics analysis reveals the response of fine roots to drought stress in cotton (*Gossypium hirsutum* L.). BMC Plant. Biol..

[12] Goche T., Shargie N.G., Cummins I., Brown A.P., Chivasa S., Ngara R. (2020). Comparative physiological and root proteome analyses of two sorghum varieties responding to water limitation. Sci. Rep..

[13] Gupta S., Mishra S.K., Misra S., Pandey V., Agrawal L., Nautiyal C.S., Chauhan P.S. (2020). Revealing the complexity of protein abundance in chickpea root under drought-stress using a comparative proteomics approach. Plant. Physiol. Biochem..

[14] Azri W., Cosette P., Guillou C., Rabhi M., Nasr Z., Mliki A. (2020). Physiological and proteomic responses to drought stress in leaves of two wild grapevines (*Vitis sylvestris*): A comparative study. Plant. Growth Regul..

[15] Zhu D., Luo F., Zou R., Liu J., Yan Y. (2021). Integrated physiological and chloroplast proteome analysis of wheat seedling leaves under salt and osmotic stresses. J. Proteomics.

[16] Amnan M.A.M., Pua T.-L., Lau S.-E., Tan B.C., Yamaguchi H., Hitachi K., Tsuchida K., Komatsu S. (2021). Osmotic stress in banana is relieved by exogenous nitric oxide. PeerJ.

[17] Ghasemzadeh A., Jaafar H.Z. (2013). Profiling of phenolic compounds and their antioxidant and anticancer activities in pandan (*Pandanus amaryllifolius* Roxb.) extracts from different locations of Malaysia. BMC Complement. Altern. Med..

[18] Reshidan N.H., Abd Muid S., Mamikutty N. (2019). The effects of Pandanus amaryllifolius (Roxb.) leaf water extracts on fructose-induced metabolic syndrome rat model. BMC Complement. Altern. Med..

[19] Agroforestry.org. https://agroforestry.org/images/pdfs/P.tectorius-pandanus.pdf.

[20] Gurmeet S., Amrita P. (2015). Unique Pandanus—Flavour, Food and Medicine. J. Pharma. Phytochem..

[21] Kumar S., Sachdeva S., Bhat K.V., Vats S., Vats S. (2018). Plant responses to drought stress: Physiological, biochemical and molecular basis. Biotic Abiotic Stress Tolerance in Plants.

[22] Reyes J.A.O., Carpentero A.S., Santos P.J.A., Delfin E.F. (2020). Effects of water regime, genotype, and formative stages on the agro-physiological response of sugarcane (*Saccharum officinarum* L.) to drought. Plants.

[23] Júnior S.D.O., de Andrade J.R., dos Santos C.M., Silva A.L.J., Endres L., Silva J.V., dos Santos Silva L.K. (2020). Osmoregulators’ accumulation minimizes the effects of drought stress in sugarcane and contributes to the recovery of photochemical efficiency in photosystem II after rewatering. Acta. Physiol. Plant..

[24] Zhang F., Zhu K., Wang Y.Q., Zhang Z.P., Lu F., Yu H.Q., Zou J.Q. (2019). Changes in photosynthetic and chlorophyll fluorescence characteristics of sorghum under drought and waterlogging stress. Photosynthetica.

[25] Sanjari S., Shobbar Z.-S., Ghanati F., Afshari-Behbahanizadeh S., Farajpour M., Jokar M., Khazaei A., Shahbazi M. (2021). Molecular, chemical, and physiological analyses of sorghum leaf wax under post-flowering drought stress. Plant. Physiol. Biochem..

[26] Oraee A., Tehranifar A. (2020). Evaluating the potential drought tolerance of pansy through its physiological and biochemical responses to drought and recovery periods. Sci. Hortic..

[27] Dinç E., Ceppi M.G., Tóth S.Z., Bottka S., Schansker G. (2012). The chl a fluorescence intensity is remarkably insensitive to changes in the chlorophyll content of the leaf as long as the chl a/b ratio remains unaffected. Biochim. Biophys. Acta (BBA)-Bioenerg..

[28] Yang F., Feng L., Liu Q., Wu X., Fan Y., Raza M.A., Cheng Y., Chen J., Wang X., Yong T. (2018). Effect of interactions between light intensity and red-to-far-red ratio on the photosynthesis of soybean leaves under shade condition. Environ. Exp. Bot..

[29] Lau S.E., Hamdan M.F., Pua T.L., Saidi N.B., Tan B.C. (2021). Plant nitric oxide signaling under drought stress. Plants.

[30] Fiasconaro M.L., Lovato M.E., Antolín M.C., Clementi L.A., Torres N., Gervasio S., Martín C.A. (2019). Role of proline accumulation on fruit quality of pepper (*Capsicum annuum* L.) grown with a K-Rich compost under drought conditions. Sci. Hortic..

[31] Liu Y., He Z., Xie Y., Su L., Zhang R., Wang H., Li C., Long S. (2021). Drought resistance mechanisms of *Phedimus aizoon* L.. Sci. Rep..

[32] Marcińska I., Czyczyło-Mysza I., Skrzypek E., Filek M., Grzesiak S., Grzesiak M.T., Janowiak F., Hura T., Dziurka M., Dziurka K. (2013). Impact of osmotic stress on physiological and biochemical characteristics in drought-susceptible and drought-resistant wheat genotypes. Acta. Physiol. Plant..

[33] Hossain M.A., Hoque M.A., Burritt D.J., Fujita M., Ahmad P. (2014). Proline protects plants against abiotic oxidative stress: Biochemical and molecular mechanisms. Oxidative Damage to Plants.

[34] Raja V., Qadir S.U., Alyemeni M.N., Ahmad P. (2020). Impact of drought and heat stress individually and in combination on physio-biochemical parameters, antioxidant responses, and gene expression in *Solanum lycopersicum*. 3 Biotech.

[35] Nagaraju M., Kumar A., Rajasheker G., Manohar Rao D., Kavi Kishor P.B. (2020). DnaJs, the critical drivers of Hsp70s: Genome-wide screening, characterization, and expression of DnaJ family genes in *Sorghum bicolor*. Mol. Biol. Rep..

[36] Carmo L.S.T., Martins A.C.Q., Martins C.C.C., Passos M.A.S., Silva L.P., Araujo A.C.G., Brasileiro A.C.M., Miller R.N.G., Guimarães P.M., Mehta A. (2019). comparative proteomics and gene expression analysis in Arachis duranensis reveal stress response proteins associated to drought tolerance. J. Proteom..

[37] Wang T., Ye C., Wang M., Chu G. (2017). Identification of cold-stress responsive proteins in Anabasis aphylla seedlings via the ITRAQ proteomics technique. J. Plant. Interact..

[38] Zhou W., Zhou T., Li M.-X., Zhao C.-L., Jia N., Wang X.-X., Sun Y.-Z., Li G.-L., Xu M., Zhou R.-G. (2012). The Arabidopsis j-protein atdjb1 facilitates thermotolerance by protecting cells against heat-induced oxidative damage. New Phytol..

[39] Sirohi P., Yadav B.S., Afzal S., Mani A., Singh N.K. (2020). Identification of drought stress-responsive genes in rice (*Oryza sativa*) by meta-analysis of microarray data. J. Genet..

[40] Luo Y., Fang B., Wang W., Yang Y., Rao L., Zhang C. (2019). Genome-wide analysis of the rice j-protein family: Identification, genomic organization, and expression profiles under multiple stresses. 3 Biotech.

[41] Pandey A., Chakraborty S., Datta A., Chakraborty N. (2008). Proteomics approach to identify dehydration responsive nuclear proteins from chickpea (*Cicer arietinum* L.). Mol. Cell. Proteom..

[42] Choudhary M.K., Basu D., Datta A., Chakraborty N., Chakraborty S. (2009). Dehydration-Responsive nuclear proteome of rice (*Oryza sativa* L.) illustrates protein network, novel regulators of cellular adaptation, and evolutionary perspective. Mol. Cell. Proteom..

[43] Razi K., Muneer S. (2021). Drought Stress-Induced Physiological Mechanisms, Signaling Pathways and Molecular Response of Chloroplasts in Common Vegetable Crops. Crit. Rev. Biotechnol..

[44] Rasouli F., Kiani-Pouya A., Shabala L., Li L., Tahir A., Yu M., Hedrich R., Chen Z., Wilson R., Zhang H. (2021). Salinity effects on guard cell proteome in *Chenopodium quinoa*. Int. J. Mol. Sci..

[45] Hyun T.K., van der Graaff E., Albacete A., Eom S.H., Großkinsky D.K., Böhm H., Janschek U., Rim Y., Ali W.W., Kim S.Y. (2014). The Arabidopsis PLAT domain protein1 is critically involved in abiotic stress tolerance. PLoS ONE.

[46] Li J., Blanchoin L., Staiger C.J. (2015). Signaling to actin stochastic dynamics. Annu. Rev. Plant. Biol..

[47] Li H.-M., Liu S.-D., Ge C.-W., Zhang X.-M., Zhang S.-P., Chen J., Shen Q., Ju F.-Y., Yang Y.-F., Li Y. (2019). Association analysis of drought tolerance and associated traits in upland cotton at the seedling stage. Int. J. Mol. Sci..

[48] Hawkins T.J., Deeks M.J., Wang P., Hussey P.J. (2014). The evolution of the actin binding NET superfamily. Front. Plant. Sci..

[49] Saha B.C. (2000). α-l-Arabinofuranosidases: Biochemistry, molecular biology and application in biotechnology. Biotechnol. Adv..

[50] Zhu J., Alvarez S., Marsh E.L., LeNoble M.E., Cho I.-J., Sivaguru M., Chen S., Nguyen H.T., Wu Y., Schachtman D.P. (2007). Cell wall proteome in the maize primary root elongation zone. ii. region-specific changes in water soluble and lightly ionically bound proteins under water deficit. Plant. Physiol..

[51] López-Hinojosa M., de María N., Guevara M.A., Vélez M.D., Cabezas J.A., Díaz L.M., Mancha J.A., Pizarro A., Manjarrez L.F., Collada C. (2021). rootstock effects on scion gene expression in maritime pine. Sci. Rep..

[52] Nemati M., Piro A., Norouzi M., Moghaddam Vahed M., Nisticò D.M., Mazzuca S. (2019). Comparative physiological and leaf proteomic analyses revealed the tolerant and sensitive traits to drought stress in two wheat parental lines and their F6 progenies. Environ. Exp. Bot..

[53] Yang J., Zhang J., Li C., Zhang Z., Ma F., Li M. (2019). Response of sugar metabolism in apple leaves subjected to short-term drought stress. Plant. Physiol. Biochem..

[54] La V.H., Lee B.-R., Islam M.T., Park S.-H., Lee H., Bae D.-W., Kim T.-H. (2019). Antagonistic shifting from abscisic acid- to salicylic acid-mediated sucrose accumulation contributes to drought tolerance in *Brassica napus*. Environ. Exp. Bot..

[55] Turner N.C. (1981). Techniques and experimental approaches for the measurement of plant water Status. Plant. Soil.

[56] Quan W., Hu Y., Mu Z., Shi H., Chan Z. (2018). Overexpression of AtPYL5 under the control of guard cell specific promoter improves drought stress tolerance in arabidopsis. Plant. Physiol. Biochem..

[57] Schneider C.A., Rasband W.S., Eliceiri K.W. (2012). NIH Image to ImageJ: 25 years of image analysis. Nat. Methods.

[58] Bresson J., Bieker S., Riester L., Doll J., Zentgraf U. (2018). A guideline for leaf senescence analyses: From quantification to physiological and molecular investigations. J. Exp. Bot..

[59] Lichtenthaler H.K. (1987). Chlorophylls and carotenoids: Pigments of photosynthetic biomembranes. Methods in Enzymology; Plant Cell Membranes.

[60] Heath R.L., Packer L. (1968). Photoperoxidation in isolated chloroplasts: I. kinetics and stoichiometry of fatty acid peroxidation. Arch. Biochem. Biophys..

[61] Bates L.S., Waldren R.P., Teare I.D. (1973). Rapid determination of free proline for water-stress studies. Plant. Soil.

[62] Dhindsa R.S., Matowe W. (1981). Drought tolerance in two mosses: Correlated with enzymatic defence against lipid peroxidation. J. Exp. Bot..

[63] Aebi H., Bergmeyer H.U. (1974). Catalase. Methods of Enzymatic Analysis.

[64] Chen G.-X., Asada K. (1992). Inactivation of ascorbate peroxidase by thiols requires hydrogen peroxide. Plant. Cell Physiol..

[65] Chance B., Maehly A.C. (1955). Assay of catalases and peroxidases. Methods Enzymol..

[66] Mannervik B. (1999). Measurement of glutathione reductase activity. Curr. Protoc. Tox..

[67] Velikova V., Yordanov I., Edreva A. (2000). Oxidative stress and some antioxidant systems in acid rain-treated bean plants: Protective role of exogenous polyamines. Plant. Sci..

[68] Wu X., Xiong E., Wang W., Scali M., Cresti M. (2014). Universal sample preparation method integrating trichloroacetic acid/acetone precipitation with phenol extraction for crop proteomic analysis. Nat. Protoc..

[69] Bradford M.M. (1976). A rapid and sensitive method for the quantitation of microgram quantities of protein utilizing the principle of protein-dye binding. Anal. Biochem..

[70] Tyanova S., Temu T., Sinitcyn P., Carlson A., Hein M.Y., Geiger T., Mann M., Cox J. (2016). The Perseus computational platform for comprehensive analysis of (prote)omics data. Nat. Methods.

[71] Vizcaíno J.A., Côté R.G., Csordas A., Dianes J.A., Fabregat A., Foster J.M., Griss J., Alpi E., Birim M., Contell J. (2013). The PRoteomics IDEntifications (PRIDE) database and associated tools: Status in 2013. Nucleic Acids Res..

