# Supplementary material for: Drought Stress Induces Morpho-Physiological and Proteome Changes of Pandanus amaryllifolius
Source: Plants (Basel). 2022 Jan 15;11(2):221. doi: 10.3390/plants11020221 (PMC8778612; doi:10.3390/plants11020221)
Supplement: Supplementary file 1 [file plants-11-00221-s001.zip › plants-1548873-supplementary.pdf]

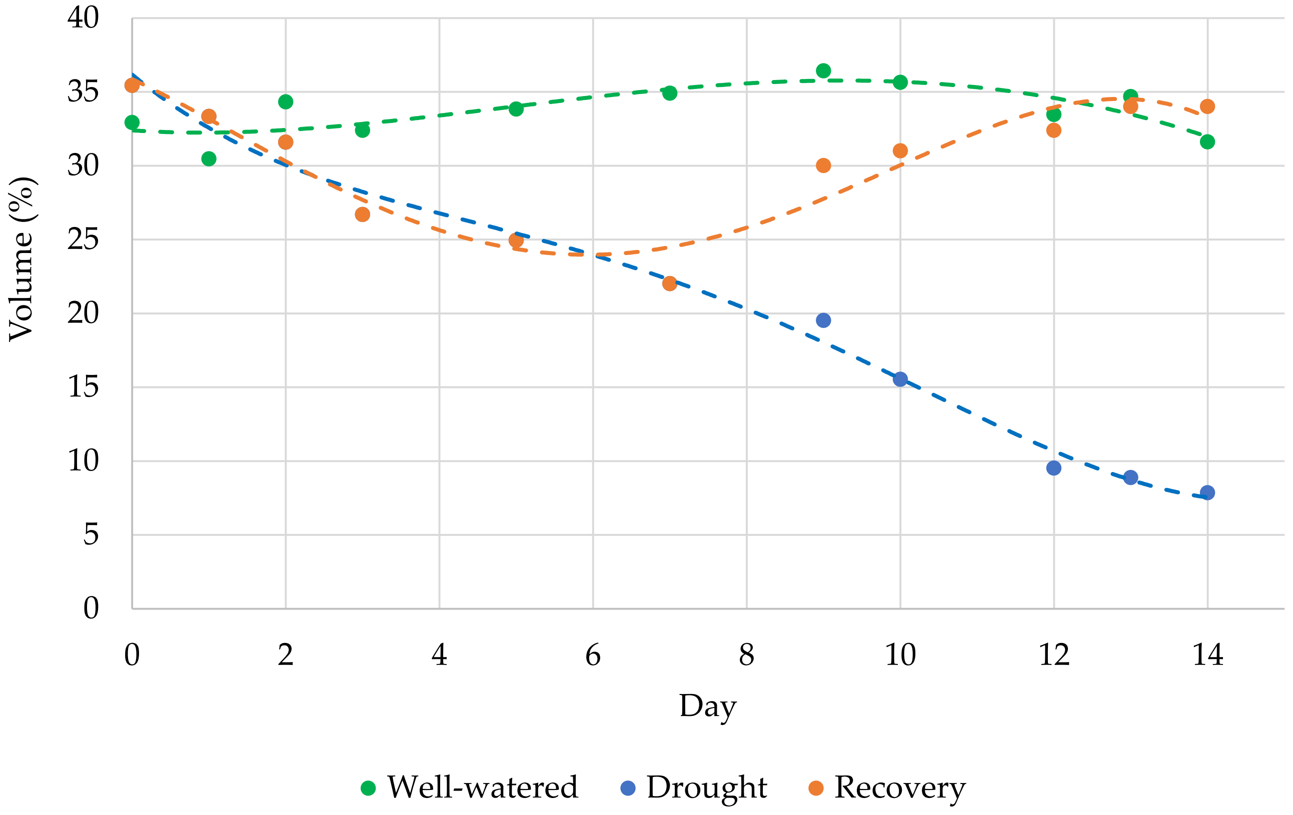

Figure S1. Soil moisture content measured in well-watered, drought and recovery samples throughout the 14 days of experiment.

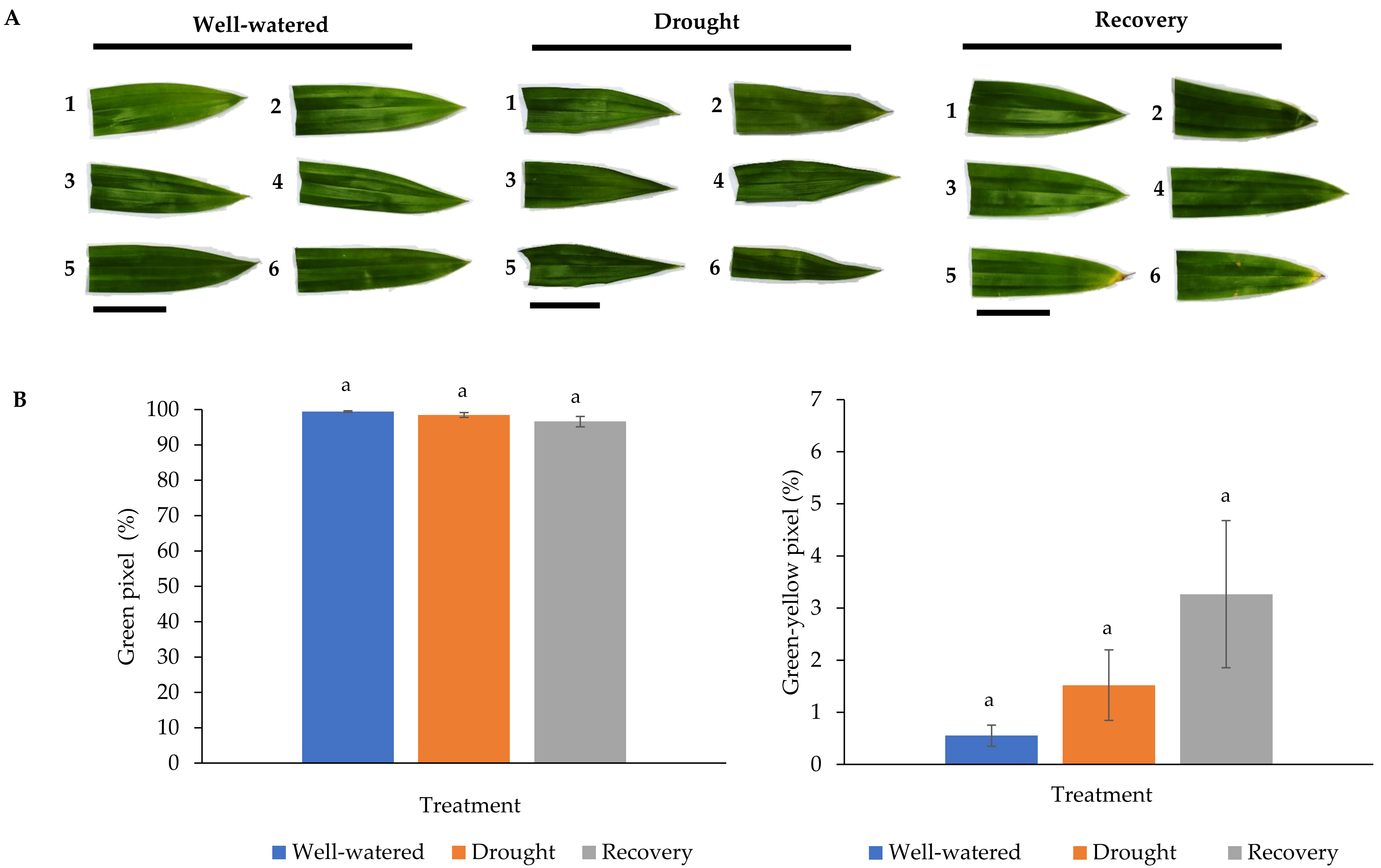

Figure S2. Comparison of *Pandanus amaryllifolius* leaves under different treatment. (**A**) The leaves were arranged according to their position from the center (number 1) to the mature leaf (number 6) and the line bar = 5 cm. This layout was used for automated colorimetric assay (ACA); (**B**) The comparison of green and green-yellow pigments pixel percentage on each time point using ACA. Means labelled with alphabet was significantly different based on the ANOVA followed by post hoc when its p-value < 0.01.

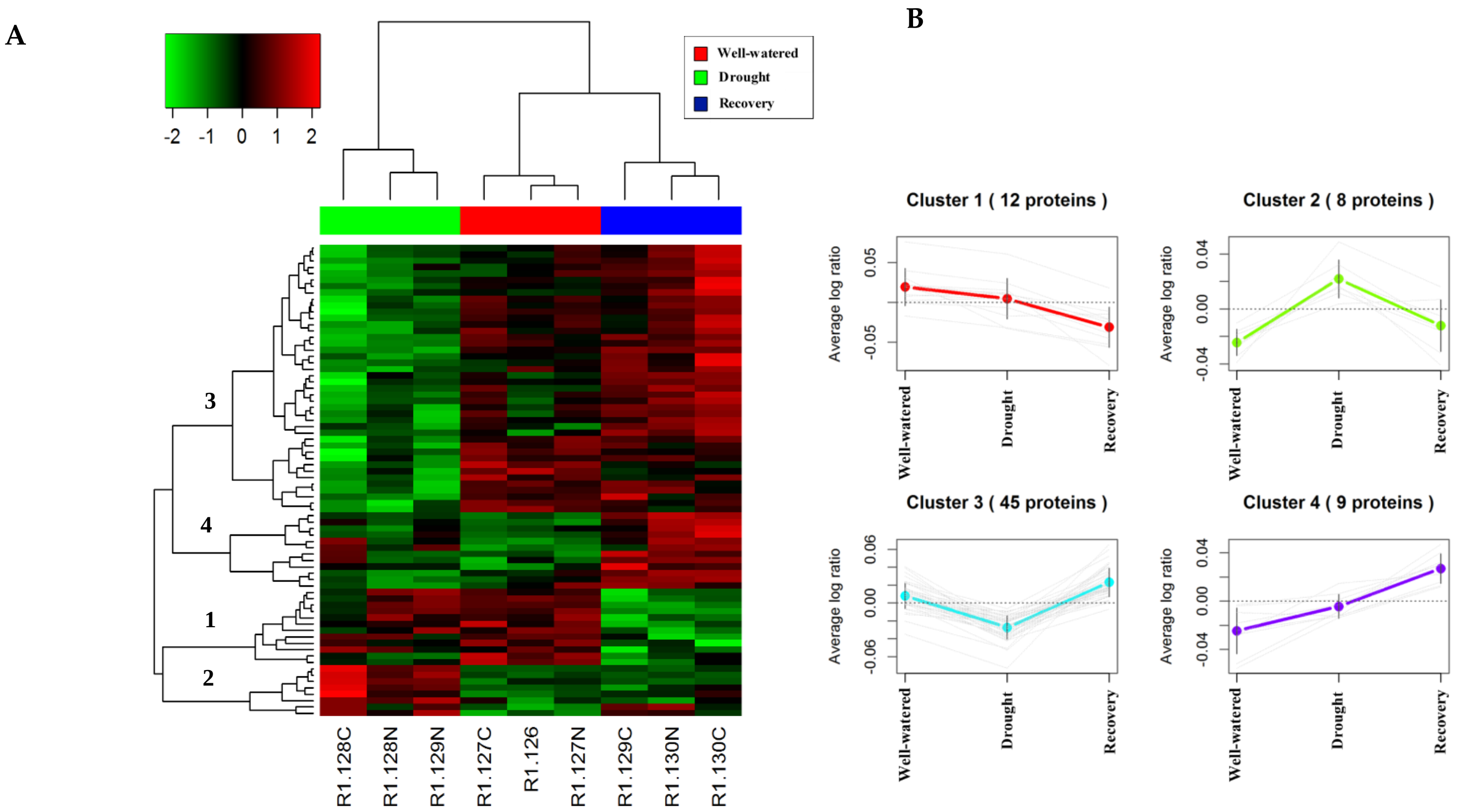

Figure S3. Hierarchal clustering of average protein abundance and clustering between treatment comparisons for well-watered, drought stressed, and recovered *Pandanus amaryllifolius*. **(A)** Heatmap of the significant protein abundance comparison between treatments according to its cluster group. The intensity scale indicates the range of upregulation (red) or downregulation (green) of proteins between treatments; **(B)** Significant differentially abundant proteins between treatments clustered into 4 groups according to the log ratio expression.

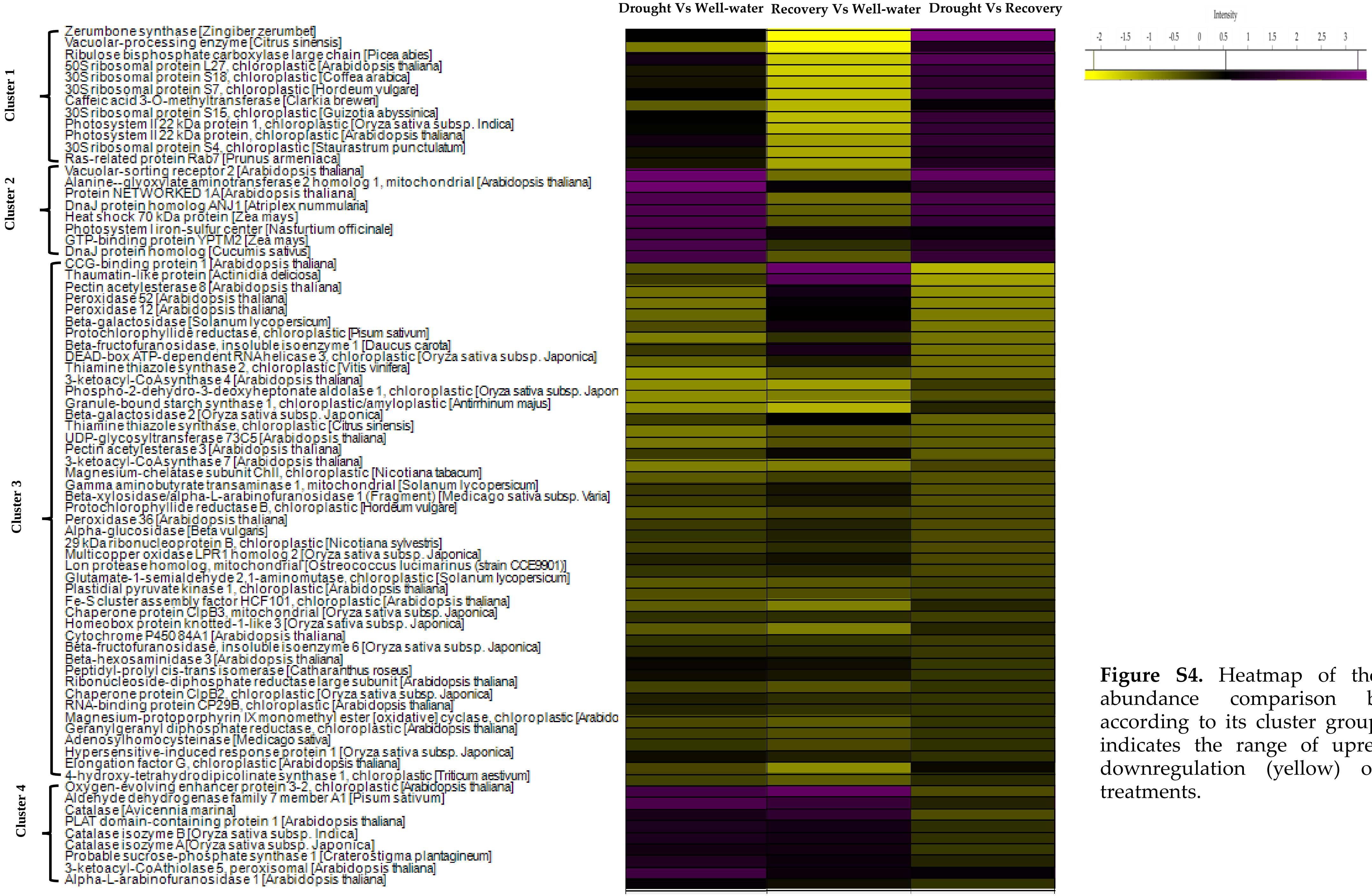

Figure S4. Heatmap of the significant protein abundance comparison between treatments according to its cluster group. The intensity scale indicates the range of upregulation (purple) or downregulation (yellow) of proteins between treatments.
